# Pilot Study on Alcohol-Induced Chemonecrosis of Hepatic Metastases From Colonic Cancer

**DOI:** 10.1155/1993/45467

**Published:** 1993

**Authors:** Aws S. Salim

**Affiliations:** 1 University Department of Surgery The Medical City Baghdad Iraq; 2 Department of Surgery National University of Malaysia 50300 Jalan Raja Muda Abdul Aziz Kuala Lumpur Malaysia

## Abstract

Administration of 98% ethanol destroys tissues by coagulative necrosis. In the rat bearing 1,2-dimethylhydrazine-induced colonic carcinoma which has spread to the liver, direct injection of 0.1–0.2 ml ethanol into each of the hepatic metastases at the time of total colectomy afforded a significant survival advantage relative to colectomy alone (20.1 ± 0.2 vs 12.8 ± 0.2 months of age, mean ± SEM, *n* = 20, *p* < 0.01 by the Mann-Whitney U test). A pilot study was, therefore, carried out (2 women and 4 men, age range 43 to 71 years—mean 56) to examine the clinical significance of these observations in patients with multiple hepatic metastases from carcinoma of the sigmoid colon. The tumour was resected then all palpable hepatic secondaries were injected with 1–1.5 ml of 98% ethanol. Two weeks post-operatively and thereafter once every two months any hepatic lesions detected ultrasonically were similarly treated percutaneously. All the patients tolerated this treatment without any observed distress or adverse effects. Their mean survival measured from the time of tumour resection until death from any cause was 20 months (range 17 to 26 months). The survival gain afforded by chemonecrosis in addition to its simplicity and safety deserves further consideration to assess the exact role of this method in the treatment of liver metastases from colonic cancer.


*HPB Surgery*, 1993, Vol. 7, pp. 33–41
Reprints available directly from the publisher
Photocopying permitted by license only

© 1993 Harwood Academic Publishers GmbH
Printed in the United States of America

### PILOT STUDY ON ALCOHOL-INDUCED CHEMONECROSIS OF HEPATIC METASTASES FROM COLONIC CANCER

### A New Approach for Percutaneous Localized Dynamic Destruction of the Hepatic Spread

AWS S. SALIM
*University Department of Surgery, The Medical City, Baghdad, Iraq*

*(Received 10 September 1992)*

Administration of 98% ethanol destroys tissues by coagulative necrosis. In the rat bearing 1,2-dimethylhydrazine-induced colonic carcinoma which has spread to the liver, direct injection of 0.1–0.2 ml ethanol into each of the hepatic metastases at the time of total colectomy afforded a significant survival advantage relative to colectomy alone (20.1 ± 0.2 vs 12.8 ± 0.2 months of age, mean ± SEM, $n = 20, p < 0.01$ by the Mann-Whitney U test). A pilot study was, therefore, carried out (2 women and 4 men, age range 43 to 71 years — mean 56) to examine the clinical significance of these observations in patients with multiple hepatic metastases from carcinoma of the sigmoid colon. The tumour was resected then all palpable hepatic secondaries were injected with 1–1.5 ml of 98% ethanol. Two weeks post-operatively and thereafter once every two months any hepatic lesions detected ultrasonically were similarly treated percutaneously. All the patients tolerated this treatment without any observed distress or adverse effects. Their mean survival measured from the time of tumour resection until death from any cause was 20 months (range 17 to 26 months). The survival gain afforded by chemonecrosis in addition to its simplicity and safety deserves further consideration to assess the exact role of this method in the treatment of liver metastases from colonic cancer.

KEY WORDS: Ethanol, chemonecrosis, colonic cancer, metastases

### INTRODUCTION

Carcinoma of the large bowel is the second leading cause of death from cancer in Western industrial countries. The most important prognostic factor for long-term survival of patients is the extent of tumour at the time of diagnosis. The high incidence and mortality rates of colonic cancer stress the need for improved therapeutic modalities that can combat this disease more effectively.

Liver metastases are present on initial diagnosis of large bowel cancer in 25 to 30% of patients[1–3]. Even after curative resection of the primary colorectal tumour between 40 and 60% of patients will develop recurrence and about 20 to 25% of them will have only liver metastases[3,4]. Once hepatic secondaries have developed,

Address correspondence to: Aws S. Salim, Department of Surgery, National University of Malaysia, 50300 Jalan Raja Muda Abdul Aziz, Kuala Lumpur, Malaysia.

the prognosis is poor, with an expected median survival time of only a few months[1]. A review from the Mayo clinic showed that 20% of patients with solitary liver metastases from colorectal cancer survived more than 3 years without treatment[4,5]. In another study, patients with synchronous solitary metastases of colorectal cancer had a 3-year survival rate of 17% compared to 1% for patients with multiple metastases[6]. It is possible that since the stage of the disease affects the outcome, early detection improves survival. Although isolated metastases exclusively confined to the liver are infrequent, a loco-regional treatment has the best chance of an effective palliation or even cure when applied to patients with metastatic spread confined to the liver. Liver resection is the only treatment which has the potential to cure a patient with liver tumours, however there seems to be an agreement to exclude patients with 4 or more metastases, extrahepatic disease, more than 50% hepatic replacement by tumour and lesions that cannot be resected with at least a margin of 10 mm[7]. Regrettably, the number of patients who fulfill these qualifications is limited and only a small number will readily benefit from liver resection[7].

Chemotherapy of liver tumours has evolved from systemic to intra-arterial treatment with single drug to multiple drug combinations and to the innovative approach with delivery systems like the implantable pump for continuous administration of the treatment. The most consistently used chemotherapeutic drug has been 5-FU. Systemic administration of this agent for the treatment of liver metastases from colorectal cancer induces an objective response in between 15 and 25% of patients yet no significant survival gain has been reported[8]. On the other hand, higher response was noted when chemotherapeutic medicines, particularly fluorodeoxyuridine, were infused intra-arterially into the liver[9,10]. It is rather disheartening to note that complications such as chemical hepatitis, gastritis, duodenitis, cholecystitis, thrombosis of the arterial or venous line, and biliary sclerosis are not uncommon and lead to the cessation of treatment. Until chemotherapeutic regimens can be developed that significantly improve the survival of patients with advanced colonic cancer and can be expected to cause objective tumour shrinkage in at least half of the patients being treated, the use of these drugs can only afford limited gains.

Interruption of the hepatic arterial circulation by simple ligation of the hepatic artery, ligation with permanent or intermittent dearterialization and embolization with non-degradable or degradable material have all been clinically employed to produce necrosis of hepatic metastases from colorectal cancer. These procedures are not specifically directed at the metastases and may produce such complications as liver abscesses, liver failure, haemorrhage and septicaemia. Moreover, although considerable tumour regression may occur after hepatic artery ligation and complete dearterialization, survival seems not to significantly increase[11]. This was thought to be due to survival of neoplastic cells on the tumours' rim. Consequently, arterial interruption was combined with infusion of cytotoxic agents to attack the surviving fraction on the rim[12]. However, the complication rate was rather high[12]. Repeated intermittent dearterialization in an attempt to confine damage to the liver metastases and to reduce injury to normal hepatic tissue while combating the tendency for arterial collateral formation may overcome some of the limitations of current methods of interrupting the hepatic arterial circulation[13].

Other modalities for the treatment of hepatic metastases are also available, but they all have limitations. The efficacy of regional or total body hyperthermia depends on tumour size and variations in blood flow which have a major influence on heat dissipation and precludes its uniform delivery. Techniques employing ultrasound and microwave heating are more commonly used but are mainly restricted to superficial tumours[14]. Devitalization of hepatic tumours by cryotherapy imposes the difficulty of adjusting the volume of frozen tissue and restricting it to the tumour[15]. Furthermore, the frozen mass per unit time is a function of freezing duration and blood supply so that a considerable time may be needed depending on the size and blood flow of a particular tumour to achieve a complete freezing[15].

It is consequently construed that efforts should not be spared in the search for an effective and simple yet safe method which enables localized destruction of hepatic metastases without any significant damage to the surrounding normal paranchyma or to the rest of the body's tissues and organs. On the basis of the fact that new hepatic metastases continue to appear, it is necessary to have a dynamic therapeutic system which could be repeatedly applied without any significant inconvenience or risks and this is perhaps the real challenge.

Injection of ethyl alcohol in high concentrations into tissues produces coagulative necrosis[16]. This communication reports the experimental and clinical results of injecting hepatic metastases from colonic carcinoma with alcohol.

### METHODS AND RESULTS

The benefits of direct injection of 98% ethanol (BDH Chemicals, Poole, UK) into hepatic metastases from 1,2-dimethylhydrazine (DMH)-induced colonic cancer was investigated in the Sprague-Dawley rat. At 10 weeks of age, rats were subcutaneously injected every week with 10 mg/kg DMH for 28 weeks. They were then housed for 3 months. At the end of this period, it was noticed that all the animals had developed colonic carcinoma with multiple hepatic metastases. These lesions occurred in both lobes of the liver and ranged between 5 and 12 tumours per animal (mean 7). Total colectomy and the fashioning of an ileostomy coupled with direct injection of 0.1 - 0.2 ml of ethanol into each of the hepatic metastases, after mobilizing the liver by dividing its fascial attachments to facilitate easier tumour detection by inspection and palpation, afforded a significant survival advantage relative to colectomy alone (20.1 ± 0.2 months of age, vs 12.8 ± 0.2 months of age, mean ± SEM, $n = 20, p < 0.01$ by the Mann-Whitney U Test). At autopsy, all the control animals demonstrated marked increase in the size and number of their hepatic lesions, which appear to have been the cause of death rather than extra hepatic tumour spread. The latter spread was observed in only 3 of these animals. Conversely, all the rats in the ethanol treatment group developed extra hepatic metastases, particularly in the lungs, and had liver lesions not different in number, size or pattern of distribution to those noted at the time of the injections. The impression thus gathered is that these animals had probably died from disease spread beyond the liver.

The clinical implications of these observations were, therefore, examined in a pilot study carried out at the Medical City in Iraq on six patients (2 women and 4 men with an age range of 43 to 71 years, mean 56) who had adenocarcinoma of the sigmoid colon with multiple hepatic secondaries. The study was approved by the Ethical Committee on Human Experimentation and every patient gave written informed consent. Exclusion criteria were as follows: age over 80 years; risk factors

(previous colonic cancer or adenomatous polyp(s), familial polyposis, ulcerative colitis, Crohn's disease); emergency admission or fever, septicaemia, or peritonitis on presentation; jaundice; ascites; leg oedema; liver metastases not readily palpable at laparotomy or fewer than 5 hepatic metastases; presence of extrahepatic metastases (pulmonary); pregnancy; alcoholism; taking any form of regular medication; hypertension; diabetes; serious underlying diseases; previous gastrointestinal surgery; history of radiotherapy, chemotherapy or any malignancy; undifferentiated carcinoma; synchronous carcinomas; or invasion of the abdominal wall or adjacent structures found during laparotomy. Sigmoid carcinoma was diagnosed by endoscopy with biopsies and double contrast barium enema. The liver was examined by ultrasound and carcinoembryonic antigen (CEA) levels were determined (upper limit of normal 25 $\mu$g/L). The bowel was prepared by a morning and an evening sodium picosulphate sachet on the day preceding surgery. At induction of anaesthesia, all patients were given 500 mg of ampicillin, 80 mg of gentamicin and 500 mg of metronidazole. Following exploration of the abdominal cavity, any palpable lymph nodes at the porta hepatis were excised for histologic examination and all the palpable hepatic metastases were biopsied using a Tru-cut needle. The colonic tumour was resected and an end to end anastomosis effected, then all the palpable hepatic metastases were slowly injected under direct vision with 1–1.5 ml of 98% ethanol using a 22 gauge needle. The fascial attachments of the liver were divided as deemed necessary to facilitate exposure. The number of the hepatic secondaries ranged from 5 to 14 with a mean of 9 and occurred in both lobes of the liver. Two of the patients studied had carcinoma in their porta hepatis lymph nodes. No blood transfusions were administered to any patient.

All the patients made an uneventful recovery from surgery apart from one patient who developed a wound infection. None of the patients experienced any significant discomfort or pains that could be attributed to the ethanol injections. Moreover, these injections caused no pyrexia or abnormal liver reactions reflected in the liver function tests (only marginal elevation of the aminotransferases was noted in the immediate postoperative period). The liver ultrasound scan was repeated two weeks following surgery to detect and inject any residual hepatic metastases. These lesions were found in every patient, were not more than two in number, and were injected percutaneously under ultrasound control using a long 22 gauge needle (Cook Inc., Bloomington, Indiana, USA) — the skin port of entry was anaesthetized with 1% plain xylocaine — and delivering 1- 1.5 ml of ethanol into each lesion. Ultrasonography enabled the completeness of tumour destruction to be assessed. At this stage it was noted that the serum alkaline phosphatase (normal range: 40–120 iu/L) and CEA levels were significantly ($p < 0.001$ by the Mann-Whitney U test) lower than their corresponding preoperative levels (93 iu/L $\pm$ 4.1 vs 211 iu/L $\pm$ 3.7 and 26.1 $\mu$g/L $\pm$ 1.3 vs 81.3 $\mu$g/L $\pm$ 4.8, mean $\pm$ SEM). All the patients were then reviewed every two months as out-patients when sigmoidoscopy (60 cm flexible) with biopsies of any suspicious colorectal mucosa, full blood counts, liver function tests, CEA levels, faecal testing for occult blood and a liver ultrasound scan with percutaneous injection of any obvious or suspicious lesions were carried out for the rest of the patients' lives. During each of these reviews, at least one and no more than three hepatic lesions were detected and treated in every patient. No obvious adverse effects caused by the ethanol injections were noted in any patient and all were observed to enjoy a good quality life. None of the patients died during the first postoperative year. Death was due to disease spread in all the patients. The mean survival measured from the time of tumour resection until death from any cause was 20 months (range 17 to 26 months).

### DISCUSSION

Patients with malignancies that have metastasised to the liver have a poor prognosis. In the presence of untreated synchronous hepatic metastases from colorectal carcinoma, the survival time can be as short as a few months. While in some of these patients surgical excision of the hepatic lesions can afford long-term survival[4,5,7], results with other therapeutic measures have been disappointing in this respect.

It has been reported[4] that approximately one-fourth of the patients bearing liver metastases from colorectal cancer have hepatic tumours that can be resected, but only 25% of these patients will live 5 years or more after such tumours have been removed. On the other hand, the incidence of recurrence in the liver alone following resection of hepatic metastases varies from 5 to 28%[4]. This recurrence is largely determined by the following: involved or narrow resective margins, bilaterality of resected lesions and the number of hepatic metastases ($<4$) removed. Since it is not ethical to deny patients treatment of proven value, the natural history of untreated cancer cannot be considered the standard against which the effectiveness of any treatment is measured. Within this context retrospective analyses based on historical controls are confusing and unreliable. Consequently, resective treatment being the most effective therapeutic weapon available against hepatic metastases becomes the standard against which any new treatment for these lesions can be tested.

The present study introduces chemonecrosis by alcohol as a new approach for localized dynamic destruction of hepatic metastases from colonic cancer. The results suggest that this approach offers a survival gain relative to other therapeutic modalities such as continuous intra-arterial chemotherapy, temporary dearterialization, and repeated intermittent dearterialization. In addition, chemonecrosis avoids the operative mortality of resectional surgery and also avoids its limitations be they tumour-related or operative risk-related. These advantages over resective treatment may be even more appreciated when the latter treatment is used only for palliation, because metastatic lesions in the liver cause pain, or when the patient may succumb from these lesions before being troubled by other sites of spread. Under these circumstances and even in the presence of other indicators of poor prognosis, the additive effect of palliation is a reasonable goal particularly if therapeutic success is to be interpreted in terms of the quality and comfort of the remaining part of a patient's life and not only the duration of this life. Although it has been reported[4] that in patients who had major hepatic resections the period of life without symptoms closely paralleled length of life so that patients were not made to live longer only to suffer more, chemonecrosis can avoid altogether the time and discomfort involved in major surgery in addition to being feasible in those patients considered a poor anaesthetic or surgical risk.

One of the most important points to note about chemonecrosis is that it requires no special devices or systems to be installed during surgery and can be applied whenever hepatic secondaries are noted. The advantage of this point can be readily realized when one recalls the proportion of colorectal cancer cases that do not appear to have metastasised to the liver, yet soon after their resection hepatic secondaries become obvious. Whether this last effect is produced by manipulations of the primary lesion, or represents metastases reaching the liver from affected lymphatics, or growth of metastases already present in the liver at the time of surgery to a size which allows detection, it can be effectively dealt with by percutaneous chemonecrosis. The dynamicity of this approach also means that any lesions which were missed can be treated the following session. In connection with this point the tremendous regenerative power of the liver enables these sessions to be repeated without any fear of serious consequences. In patients with colorectal cancer, exact diagnosis of the presence or absence of liver metastases is not always easy. Although it is not necessary to destroy all the hepatic secondaries during surgery for the primary lesion and may indeed be undesirable in poor risk patients or when it is thought unwise to increase operative time or extend the magnitude of surgery and increase its impact on the patient, the improved detectability of liver metastasis at the time of colorectal operation by the use of intraoperative ultrasonography overcomes the diagnostic limitations of percutaneous ultrasonography and CT scanning by detecting preoperatively unknown or nonpalpable lesions thereby helping operative decision making and providing a baseline for postoperative chemonecrosis. Considering the fact that this method is relatively simple, inexpensive and can be repeatedly applied without any apparent complications or mortality, it deserves a more detailed analysis through randomized trials to determine its exact place in the treatment of hepatic secondaries in comparison with current treatment options.

The possibility that removal of the primary tumour accelerates the growth of micrometastases may explain the clinical observation that control of hepatic metastases stimulates tumour growth at extrahepatic sites[13]. It can, however, be argued that this control allows time for extra hepatic tumour sites to occur which would not have otherwise been encountered due to the limited survival. Tumours at these sites may be more readily detected by CT scanning and it is perhaps very much worthwhile from a survival point of view to consider destroying them percutaneously by chemonecrosis.

*Acknowledgements*

I am grateful to Dr S.H. Alwash for giving me the opportunity to carry out this work and I thank Mrs Jutta Gaskill for her skillful secretarial work.

*References*

1. Bengmark, S. and Hafström, L. (1969) The natural history of primary and secondary malignant tumors of the liver. 1. The prognosis for patients with hepatic metastases from colonic and rectal carcinoma by laparotomy. *Cancer*, **23**, 198–201
2. Finlay, I.G., Meek, D. and McArdle, C.S. (1981) Metastases in the liver. *Br. Med. J.*, **283**, 307–308
3. Russel, A.H., Tong, D., Dawson, L.E. and Wisbeck, W. (1984) Adenocarcinoma of the proximal colon: sites of initial dissemination and patterns of recurrence following surgery alone. *Cancer*, **53**, 360–367
4. Adson, M.A. (1987) Resection of liver metastases — when is it worthwhile? *World J. Surg.*, **11**, 511–520

5. Wagner, J.S., Adson, M.A., Van Heerden, J.A., Adson, M.H. and Ilstrup, D.M. (1984) The natural history of hepatic metastases from colorectal cancer. A comparison with resective treatment. *Ann. Surg.*, **199**, 502–507
6. Wanebo, H.J., Semoglar, C., Attiyeh, F. and Stearns, M.J. (1978) Surgical management of patients with primary operable colorectal cancer and synchronous liver metastases. *Am. J. Surg.*, **135**, 81–84
7. Adson, M.A., Van Heerden, J.A., Adson, M.H., Wagner, J.S. and Ilstrup, D.M. (1984) Resection of hepatic metastases from colorectal cancer. *Arch. Surg.*, **119**, 647–651
8. Kemeny, N. (1983) The systemic chemotherapy of hepatic metastases. *Semin. Oncol.*, **10**, 148–158
9. Kemeny, N., Reichman, B., Oderman, P., Daly, J. and Geller, N. (1986) Update of randomized study of intrahepatic vs systemic infusion of fluorodeoxyuridine (FUDR) in patients with liver metastases from colorectal carcinoma. *Proc. Am. Soc. Clin. Oncol.*, **5**, 345
10. Kemeny, N. and Daly, J. (1988) Preliminary results of a randomized study of intra-hepatic infusion versus systemic infusion of 5-Fluoro-2-deoxyuridine for metastatic colorectal carcinoma. *Recent Results in Cancer Research*, **100**, 171–177
11. Almersjö, O., Bengmark, S., Rudenstam, C.M., Hafström, L-O and Nilsson, L.A.V. (1972) Evaluation of hepatic dearterialization in primary and secondary cancer of the liver. *Am. J. Surg.*, **124**, 5–9
12. Dahl, E.P., Fredlund, P.F., Tylen, U. and Bengmark, S. (1981) Transient hepatic dearterialization followed by regional intra-arterial 5-fluorouracil infusion as treatment for liver tumors. *Ann. Surg.*, **193**, 82–88
13. Persson, B.G. (1990) Repeated intermittent dearterialization in the treatment of liver tumors. An experimental and clinical study. *Bull.*, **79**, Lund University, Sweden
14. Gerweck, L.E. (1985) Hyperthermia in cancer therapy: the biological basis and unresolved questions. *Cancer Res.*, **45**, 3408–3414
15. Charnley, R.M., Doran, J. and Morris, D.L. (1989) Cryotherapy for liver metastases: a new approach. *Br. J. Surg.*, **76**, 1040–1041
16. Salim, A.S. (1991) Surgery or chemoneurolysis for complete vagal denervation of rat stomach? *Dig. Dis. Sci.*, **36**, 1074–1078

*(Accepted by S. Bengmark 10 September 1992)*

### COMMENTARY

Liver metastases are the main cause of death in patients with colorectal cancer. A fact that is particularly relevant in the United States where over 150,000 people a year develop colon cancer and over 60,000 die of it. Since the liver is the main site of metastatic spread any remedy for liver metastases is needed and usually eagerly adopted.

Dr Salim's study is a report on a new method for treating liver metastases, the direct injection of absolute alcohol into liver tumors. He reports on his laboratory work as well as on a pilot study in 6 patients. In his laboratory study 20 rats were placed into a treatment or control group. All animals had chemically induced colon cancer and had multiple liver metastases. All animals were treated with a colectomy. The treatment group also received multiple injections of ethanol into the liver metastases. The rats in the control group without the injections died at 12.8 months while those with the ethanol died at 20.1 months. The control animals all had increased liver metastases at death while the treatment group seemed to have no progression in their liver metastases but had extrahepatic spread.

This laboratory experience seems very similar to the clinical effects that have been seen in patients with hepatic metastases from colorectal primaries who are treated with continuous hepatic artery infusions (CHAI) of floxuridine (FUDR). Most of the studies on the FUDR infusions have shown increased hepatic control with modest survival benefits[1,2], but eventual demise of the patients because of extrahepatic disease. One additional fact known about FUDR infusions is the objective response rate which in selected patients can be as high as 93%[3]. Dr Salim did not tell us about the objective response in the rats so I would assume their was no response but only stable disease.

From this limited success he went on to do a pilot study in 6 patients. The patients all had primary colon cancers that had not yet been resected. All patients underwent a colon resection and injection of hepatic metastases at the same operation. Two weeks after surgery the patients had percutaneous injections of any ultrasonographically apparent lesions. He mentioned that the CEA and alkaline phosphatase went down after the operations. The significance of this is questionable since all patients had their liver metastases. Over the ensuing months these patients were followed and each time a liver metastases turned up it was treated with percutaneous injections. The mean survival time for these 6 patients was 20 months (17–26 months).

Is this an exciting report? It is hard to say at this time. It seems that the 6 patients who received the treatment had a prolonged survival with a mean survival of 20 months, but it is hard to know in select patients if this really means anything. Certainly compared to no treatment it is an improvement. However, in a study I carried out at the City of Hope when selected patients with multiple liver metastases were treated with CHAI of FUDR by the implantable Infusaid pump the mean survival was 19.8 months[3]. Furthermore 93% partial or complete hepatic responses were seen in this group. As in the Iraqi study most patients died from extra-hepatic disease.

Dr Salim's study does not address response rates and it suggests that all the patients had recurrent hepatic metastases. In the City of Hope study we did not see recurrent hepatic disease. I certainly think that the ethanol injections have not yet been shown to be superior to CHAI (even though Dr Salim suggests they are). This does not mean it is not useful or interesting. One aspect that seems appealing is the apparent low toxicity. Also ethanol injections would be easy to perform in most hospital settings at a reasonable cost.

Perhaps CHAI or FUDR and ethanol injections can be combined to increase response and increase hepatic control. Certainly an approach such as the ethanol injections might and should be compared to infusion alone in a controlled study.

New modalities such as ethanol injections may not be the cure we are all looking for but in conjunction with other already adopted modalities we may be able to begin to accomplish the complete and durable responses toward which we are all working.

M. Margaret Kemeny, M.D. F.A.C.S.

Chief, Surgical Oncology

Associate Professor of Surgery

New York Medical College

### *References*

1. Rougier, P., Laplanche, A., Huguier, M. *et al.* (1992) Hepatic arterial infusion of floxuridine in patients with liver metastases from colorectal carcinoma: Long-term results of a prospective randomized trial. *J. Clin. Oncol.*, **10**, 1112–1118
2. Kemeny, N., Daly, J., Reichman, B. *et al.* (1987) Intrahepatic or systemic infusion of fluorodeoxyuridine in patients with liver metastases from colorectal carcinoma. A randomized trial. *Ann. Intern. Med.*, **107**, 459–465
3. Kemeny, M.M., Goldberg, D., Beatty *et al.* (1986) Results of a prospective randomized trial of continuous regional chemotherapy and hepatic resection as treatment of hepatic metastases from colorectal primaries. *Cancer*, **57**, 492–498

